# Characterization of an Ultra-Thin Silicon Strain Gauge Exposed to Gamma Ray Irradiation

**DOI:** 10.3390/s26082514

**Published:** 2026-04-19

**Authors:** Fan Yang, Hao Liu, Masahito Takakuwa, Tomoyuki Yokota, Takao Someya, Jarred W. Fastier-Wooller, Shun Muramatsu, Michitaka Yamamoto, Kenta Murakami, Toshihiro Itoh, Seiichi Takamatsu

**Affiliations:** 1 Department of Precision Engineering, Graduate School of Engineering, The University of Tokyo, Tokyo 113-8656, Japan; yf-utokyo@g.ecc.u-tokyo.ac.jp (F.Y.);; 2 Institute of Engineering Innovation, School of Engineering, The University of Tokyo, Tokyo 113-8656, Japan; 3 Department of Electrical and Electronic Engineering and Information Systems, School of Engineering, The University of Tokyo, Tokyo 113-8656, Japan; 4 Department of Nuclear Engineering and Management, Graduate School of Engineering, The University of Tokyo, Tokyo 113-8656, Japan; 5 School of Systems Science and Industrial Engineering, Thomas J. Watson College of Engineering and Applied Science, State University of New York at Binghamton, New York, NY 13902, USA

**Keywords:** MEMS, ultra-thin silicon, piezoresistive sensor, gamma-ray irradiation

## Abstract

Microelectromechanical systems are being increasingly deployed in nuclear industry robotics, where their great sensitivity and mechanically stable silicon structures enable reliable sensing in radiation-exposed environments. An ultra-thin silicon strain gauge without an oxide substrate layer designed for robotic electronic skin is evaluated under Co-60 γ irradiation, representative of nuclear decommissioning conditions. The sensor performance is evaluated based on electrical measurements conducted before and after irradiation, focusing on cumulative radiation-induced effects. The results show that silicon strain gauge signal maintains a high linearity (R^2^ > 0.99) under strain. Across an accumulated dose range up to approximately 15 Gy, only minor variations are observed, including a resistance increase within 1.3% and a reduction in gauge factor within 5% for most specimens. The radiation-induced resistance increases and sensitivity degradation results in a maximum strain estimation error of approximately 22.5 με (≈3.5%) within the tested operating range below 700 με.

## 1. Introduction

The long-term decommissioning of the Fukushima Daiichi Nuclear Power Plant has created a continuing need for remote robotic operations in highly contaminated areas. Access to the highly contaminated reactor buildings is essential for in situ inspection, debris sampling and retrieval tasks. These tasks often occur in confined and structurally complex environments where human access is not feasible [1,2]. Therefore, such tasks in harsh radiation environments demand specialized robotic systems capable of precise manipulation and stable long-term operation.

The radiation environment inside reactor buildings, such as the boiling water reactors at the Fukushima Daiichi decommission site, is dominated by γ-ray fields, with general air dose rates of mSv/h, while localized regions reach several Sv/h. In situ electronic devices will spend many hours in such gamma ray conditions during a single mission (TEPCO; IAEA SRS-77), so that cumulative dose becomes the relevant design parameter for electronic devices. Under these circumstances, sensing elements must maintain a stable and predictable output throughout an inspection. The robots are equipped with a durable tactile sensing element that can reliably transmit mechanical interaction information, enabling precise manipulation under radiation.

Many robotic tactile skins rely on flexible organic materials [3], which degrade rapidly under ionizing radiation due to chain scission and crosslinking [4]. In parallel, the application of various microelectromechanical systems in harsh environments, such as nuclear power plants decommission site and aerospace, is rapidly increasing [5,6,7].

Among those systems, ultra-thin silicon MEMS sensors are particularly promising for nuclear robotic applications. One such application, a method based on a silicon-on-insulator (SOI) technique, fabricated a micrometer-thick silicon gauge through backside oxide layer etching and retained the top silicon layer [8]. Micrometer-thick silicon gauges offer great sensitivity, flexibility, and long-term mechanical stability under ionizing radiation, making them the ideal structure for MEMS-based robotic skin development. In conventional SOI devices, radiation sensitivity mainly arises from charge trapping in the buried oxide, which subsequently couples to the active silicon channel [9,10]. In this study, the oxide layer was removed to improve mechanical flexibility through thickness reduction. Consequently, the absence of the Si/SiO_2_ interface eliminates interface-related charge trapping in the present structure, and such effects are therefore not considered in the subsequent analysis. The study focuses on evaluating the electrical performance of the proposed device under gamma irradiation.

Literature about the radiation test on the various silicon devices was investigated [5,6,7,11,12,13,14]. However, drawing conclusions is challenging because only a few studies have examined gamma irradiation effects on silicon piezoresistive strain gauges, and available data are often limited. Typical degradation thresholds and modes for various silicon devices are summarized in Table 1. The radiation effects, including single-event effects (SEEs) [10], displacement damage, total ionizing dose (TID) [14,15,16,17], and irradiation synergistic effects (ISEs) [18] that occurred in silicon devices were extensively studied. The investigations provide important guidelines for defining radiation tolerance thresholds in microelectronics and sensors. Zhu et al. [19] developed one polycrystalline and two silicon-on-insulator (SOI) piezoresistive pressure sensors and found that a gamma dose of 23 kGy caused a slight shift (a few mV) in the offset voltages, but no degradation of the sensitivity or linearity. Holbert et al. reported that the Kulite pressure probes unexpectedly failed after being exposed to less than 10 Gy, and Endevco 7264B-500T accelerometers exhibited a voltage shift and an increased sensitivity of 2–8% [13].

However, many studies mainly address high-dose conditions and device failure and give little information on the signal change of sensing elements operated for hours in relatively low-dose-rate γ-ray fields, where devices remain functional but may undergo gradual drift in output. Such drift does not constitute failure but can impair measurement accuracy, and its magnitude and physical cause are not fully studied. It is assumed that radiation-induced defects in semiconductor materials have the same broad effect as those produced by the impurity atoms used in doping [20]. Thus, the semiconductors that are highly doped will show a very small change in resistivity for a given dose of radiation. In addition, different design parameters of the device, such as size, thickness of layers, and electrode contact, will all affect how the radiation influences the device performance.

To address these gaps, this study experimentally evaluates ultra-thin silicon piezoresistive sensors under a 0.579 Gy/h Co-60 gamma ray field representative of nuclear-decommissioning conditions. The work focuses on three key aspects of silicon strain gauge signal behavior: (i) drift of the baseline resistance under prolonged irradiation, (ii) changes in strain sensitivity quantified by the gauge factor, and (iii) preservation of the linear strain–resistance response required for reliable tactile sensing. Four-wire resistance measurements and strain calibration before and after irradiation on a stainless-steel cantilever are combined to quantify those effects. The results are used to define a practical operating window for ultra-thin silicon gauges in low-dose-rate γ environments and to discuss calibration strategies and design implications for their use as tactile sensing elements in nuclear robotic applications.

**Table 1:** Investigation on the previous studies about radiation-induced silicon-based devices’ degradation.

Previous Studies	Device	Radiation Source	Dose Level	Degradation Mode
Belwanshi et al., 2024 [5,14]	Silicon piezoresistive pressure sensor (TE 13A-250G)	Co-60 γ	3.83~27.9 Mrad (Si)	Sensitivity degradation <0.8%; offset voltage increase of 139%.
C.I. Lee et al., 1996 [7]	Silicon accelerator (Motorola XMMAS40G)	Co-60 γ	4 krad (Si)	Failure of CMOS readout circuit.
M. Mikeštíková et al., 2025 [11]	Si MD8 diodes (Itk strip)	Co-60 γ	0.5~100 krad (Si)	Surface current increases significantly at low doses, dominates total current; bulk current is stable.
Duden et al., 2024 [12]	Itk Si strip modules	Co-60 γ	11 krad (Si)	γ irradiation cured early breakdown, raising breakdown voltage >−500 V.
Keith E. Holbert [13]	Silicon piezoresistive sensor (Kulite pressure transducers)	Gamma ray	0–800 kGy	Unexpectedly failed less than 10 Gy; Sensors basically suitable when less than 10 kGy.
S. Y. Zhu et al., 2001 [19]	SOI-based silicon piezoresistor	Co-60 γ	23 kGy (H_2_O)	Offset voltage increase, no obvious degradation in linearity or sensitivity.
D. G. Marinaro et al., 2008 [21]	P-type silicon piezoresistive sensor	3.5 MeV proton	$1\times 10^{16}\;{\mathrm{cm}}^{2}$	Both ionization and non-ionization defects; resistance increase and sensitivity decrease

To compile the studies summarized in Table 1, a structured literature survey was conducted using major academic databases, including Web of Science and Google Scholar. The search was performed using keywords such as radiation effects, ionization effect, silicon-based devices, and piezoresistive sensors. The literature search covered publications from 1995 to 2025.

Studies were selected based on their relevance to radiation type, dose level, and reported degradation mechanisms in silicon-based devices. In addition to peer-reviewed journal articles, technical reports and field investigation records related to the Fukushima Daiichi nuclear accident were also considered to reflect practical operational conditions in high-radiation environments.

The reported radiation types and dose levels in Table 1 are taken directly from the references and are therefore expressed using different dose metrics, including absorbed dose in silicon (rad(Si), Gy(Si)), absorbed dose in water (Gy(H_2_O)), and particle fluence (cm^−2^). These metrics correspond to different radiation environments and measurement conventions and are not directly comparable without detailed information on radiation type, energy, and material interactions. Therefore, Table 1 provides a qualitative overview of the reported dose ranges and corresponding degradation modes in previous studies.

## 2. Method

### 2.1. Sensor Structure Design

**Figure 1:**
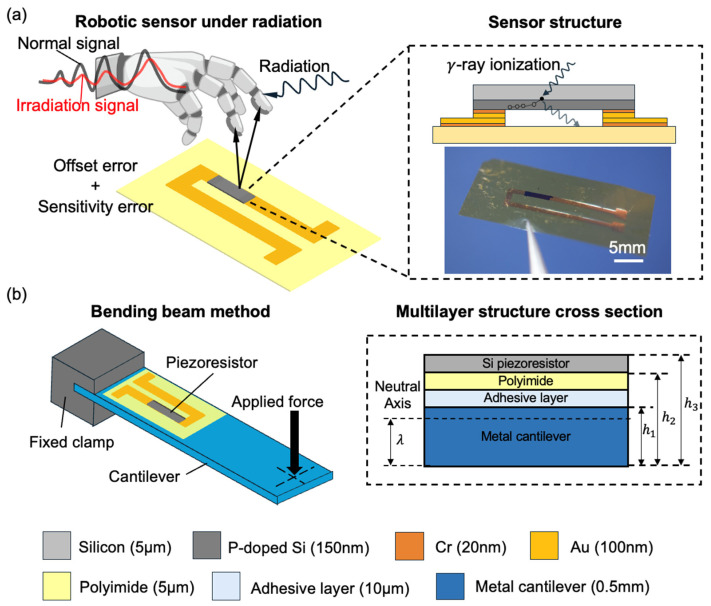
Schematic diagrams of (**a**) the application of ultra-thin silicon on the robotic skin to be used in a radiation environment and the structure of the sensor; (**b**) bending beam method for calibration of the resistivity to the strain and the multilayer structure model for calculating the strain transfer to the silicon sensors.

The sensor is designed as an ultra-thin, flexible silicon piezoresistive element to enable tactile sensing in robotic electronic skin, as shown in Figure 1a. The device was fabricated on an SOI wafer (KST World Corporation, Fukui, Japan), with a 5-μm-thick lightly doped p-type Si layer, 1-µm-thick SiO_2_ layer, and a 400-µm-thick handling bottom Si layer. The top Si layer was implanted with 150 nm deep phosphor ions and annealed at 950 °C using a lamp-annealing system to form a piezoresistive layer (150 nm). Then, the Au electrode was formed on piezoresistive layer by sputtering Au/Cr layers, followed by photolithography and etching processes. The top and bottom Si layers were patterned and etched via inductive coupled plasma reactive ion etching (ICP-RIE). The SiO_2_ layer was then etched by reactive ion etching (RIE), forming an ultra-thin silicon membrane. Finally, the Si gauge was picked up by a vacuum chip mounter (MRS-850RD, Okuhara Electric Corporation, Tokyo, Japan) using a nozzle.

Choi J.H reported a similar way successfully obtaining the ultra-thin silicon (20 μm) [22] by wet etching. In this work, the reduction of silicon thickness and the removal of the buried SiO_2_ layer are primarily intended to improve mechanical flexibility, as conventional SOI-based structures are prone to fracture under deformation. 

To facilitate the electrical connection, Cr/Au electrodes are sputtered on the silicon layer and polyimide substrate (CFS-4EP-LL, Shibaura Mechatronics Corporation, Yokohama, Japan), and the released chip is transfer-assembled onto the polyimide substrate, as shown in Figure 1a. The Au surface was activated by Aqua-plasma (Aqua Plasma Cleaner AQ-500, Samco Inc., Kyoto, Japan), and conduct direct bonding [8,23] by hands, followed by annealing at 150 °C, 10 kPa for 0.5 h using a hot plate. External interconnects are formed by Ag paste (ELEPASTE NP1, Taiyo, Ranzan, Japan) soldering, and the joints are encapsulated with PDMS (KE-1300, Shin-Etsu Chemical, Tokyo, Japan) for mechanical protection.

### 2.2. Calibration

(a) Resistance measurement

The resistance of the devices was measured before and after irradiation using a Keithley 2450 source meter operated in constant-current mode and connected to a LabVIEW-based data acquisition system at room temperature. A four-wire configuration was employed to eliminate the influence of lead and contact resistance. In this configuration, a constant bias current was supplied through the current leads, while the voltage leads measured the potential difference directly across the silicon piezoresistive element. That ensures accurate resistance determination independent of cable and contact losses. Each measurement was repeated at least five times to evaluate reproducibility and stability over time. The same instrument, cabling, and probe configuration were maintained for before and after irradiation measurements to ensure consistency. The fractional resistance change was expressed as

(1) $$∆=\frac{R-R_{0}}{R_{0}}\times 100\%$$

where R is the measured resistance and $R_{0}$ is the pristine resistance. All measurements were performed at stable room temperature using the same equipment setup.

Although resistance was monitored continuously during irradiation, temperature fluctuations affected the time-series data, so these measurements were excluded from quantitative analysis.

(b) Piezoresistive calibration

The strain gauge was mounted on a SUS304 stainless-steel I-beam (Misumi, Tokyo, Japan) using a room-temperature curing cyanoacrylate adhesive (CC-33AX5, Kyowa Electronic Instruments Co., Ltd., Tokyo, Japan). The adhesive was applied only to the load-transfer regions of the polyimide substrate to avoid constraining the sensing area. The polyimide sheet carrying the silicon gauge was then gently pressed against the prepared metal surface to remove trapped air and achieve uniform contact. After curing at 60 °C for 1 h, the adhesive formed a stable adhesive attachment suitable for cyclic tensile loading within its rated temperature range (−50 °C to 120 °C).

**Figure 2:**
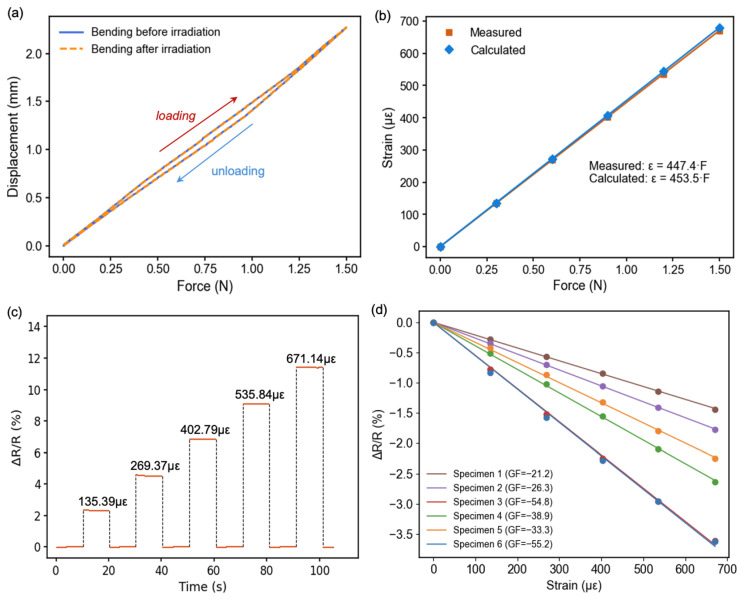
(**a**) The loading–unloading curve of the SUS304 cantilever under the applied vertical force; (**b**) strain measurement using the commercial strain gauge with the GF = 170 under five different loadings and confirmed by calculation using multilayer model; (**c**) the resistance value at each loading step was obtained by averaging the measured signal over a ten seconds holding period; (**d**) gauge factor calculation of fabricated silicon strain gauges before irradiation.

For calibration, a quasi-static loading method was employed as shown in Figure 1b. Controlled forces were applied to the free end of the cantilever in incremental steps, covering a strain range up to approximately 700 µε. During calibration, four procedures were followed to ensure data reliability. First, the loading–unloading cycles of the cantilever were carefully repeated, confirming that all force–displacement curves overlapped within experimental scatter. This verified that the same mechanical load was applied each time and that the elastic behavior of the cantilever remained unchanged throughout the tests. Figure 2a shows the loading–unloading curve before and after irradiation.

Second, to validate the accuracy of the applied strain, a commercial silicon strain gauge (KSPB-3-120-F2-11, GF = 170) was attached to the same SUS304 cantilever. And the strain was calculated from the signal of the commercial strain gauge using the equation

(2) $$\varepsilon_{measure}=\frac{∆R/R_{0}}{K_{0}}$$

where the $K_{0}$ is the gauge factor of the commercial silicon strain gauge. The theoretical strain on the surface of the SUS304 cantilever was calculated from the Euler–Bernoulli beam equation:

(3) $$\varepsilon_{theory}(x)=\frac{6F(L-x)}{Ebh^{2}}$$

where F is the applied tip force, L is the beam length, b is the beam width, h is the thickness of the SUS304 cantilever, and E is the Young’s modulus of the SUS304 cantilever. However, this classical formulation assumes a uniform elastic body, which does not hold for our multilayer structure composed of SUS304, adhesive, polyimide, and silicon layers as shown in Figure 1b. In such a system, the strain is distributed non-uniformly through the thickness, and the neutral axis (where strain = 0) shifts from the geometric center toward the stiffer substrate. To account for this, the effective neutral axis position λ was calculated as:

(4) $$\lambda =\frac{\sum_{i=1}^{n}\;E_{i}(h_{i}^{2}-h_{i-1}^{2})}{2\sum_{i=1}^{n}\;E_{i}d_{i}}$$

where $E_{i}$, $d_{i}$, and $h_{i}$ represent the Young’s modulus, layer thickness, and top coordinate of each layer, respectively. The specific parameters of each layer for calculation are recorded in Table 2.

(5) $$\varepsilon \left(y\right)=\varepsilon_{theory}\times \frac{y-\lambda }{h_{1}-\lambda }$$

where the λ is the distance of neutral layer to the bottom, $h_{1}$ is the thickness of top surface of SUS304 cantilever, and y is the distance of polyimide surface to the bottom.

As shown in Figure 2b, the measured strain–force relationship is strictly linear (R^2^ = 1), and the fitted slope (447.4 µε/N) agrees with the theoretical value (453.5 µε/N) within 1.36%, confirming the accuracy of the calibration and the validity of the neutral-axis correction. This calibration curve was used as the reference to convert force to strain in subsequent sensor tests.

Third, under each applied strain, the system was held for a fixed stabilization time to allow the electrical signal to reach equilibrium as shown in Figure 2c. The average value of the stable voltage readings was then taken as the representative resistance change for that load. This averaging minimized transient drift and improved measurement precision.

Finally, the change in resistance was obtained as shown in Figure 2d, and the measured strain was used to calculate the gauge factor of each prepared specimen. This will be used for calculating the gauge factor after irradiation.

### 2.3. Gamma Ray Irradiation Experiment

Irradiation experiments were conducted in a Cobalt 60 gamma ray facility, Takasaki Advanced Radiation Research Institute, National Institutes for Quantum Science and Technology. The Cobalt-60 source is a sealed, radioisotope produced by neutron activation of Cobalt-59, that undergoes β^−^ decay to excited Nickel-60, emitting two characteristic γ photons at 1.173 MeV and 1.333 MeV [24]. These photons constitute the primary radiation field in the Cobalt-60 irradiator. Each sealed source rod contains metallic cobalt encapsulated within double stainless-steel tubes for containment and mechanical safety.

**Figure 3:**
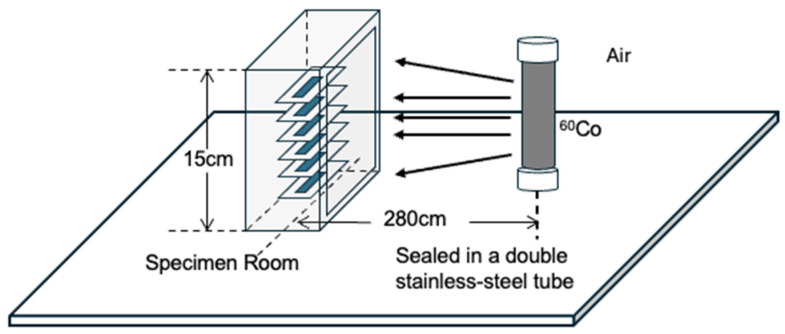
The experiment configuration in the gamma ray irradiation room.

**Figure 4:**
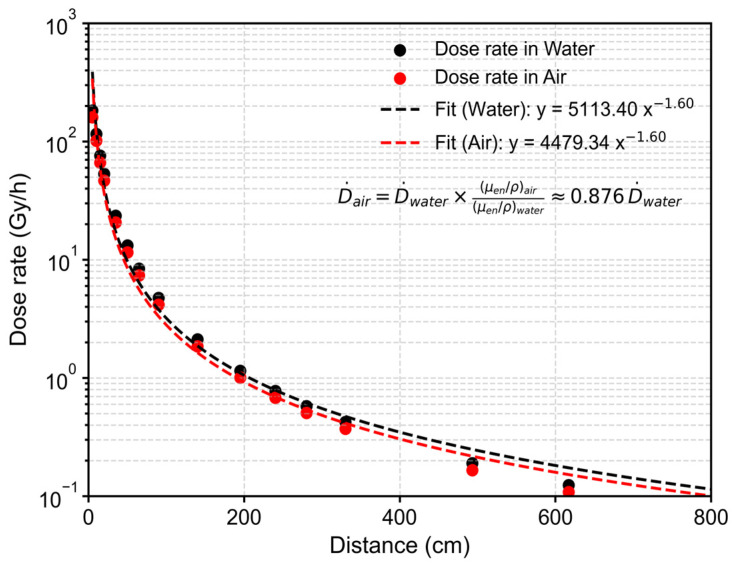
Distance–dose rate profile in the Cobalt-60 irradiation room. The horizontal axis is the distance from the source shielding plate, and the vertical axis is the absorbed dose rate to water and air. Measurements were taken at a height of 22.5 cm.

All samples were arranged along a single vertical line in the specimen room at 280 cm from the source, as shown in Figure 3. Because the separation between the top and bottom specimens is small compared with the source distance r, the dose rate difference across the stack is negligible. The relative change introduced by the inverse-square law is approximately $H^{2}/{4r}^{2}$, which is below 1%. Thus, the radiation field can be regarded as nearly uniform over the specimen room. The irradiation field was quantified in terms of dose rate to water ($D_{w}$) obtained from Takasaki Institute, as shown in Figure 4. The distance of 280 cm corresponds to a facility calibration point of 0.579 Gy h^−1^ to the water, which is approximately 0.5 Gy h^−1^ in the air. This distance provides a practical compromise between field uniformity and the total dose achievable within the available irradiation time. In addition, the gamma ray attenuation in the air is very low, so the calibrated dose rate could be directly applied to the samples without significant correction.

In this study, the irradiation conditions were designed to reproduce an environment with a dose rate comparable to that encountered during nuclear power plant decommissioning. To examine the stability of silicon-based sensor resistance under continuous γ-ray irradiation, five irradiation exposure times were selected, corresponding to total doses of approximately 0.465, 1.572, 2.781, 9.001, and 14.565 Gy (air). These exposure intervals allowed the identification of the onset dose at which resistance changes begin to appear during prolonged sensor operation under γ irradiation.

Regarding temperature conditions, the radiation room was air-conditioned during operation, targeting approximately 25 °C. The temperature fluctuations about ±2~3 °C were possible due to periodic air-conditioning cycling. Such temperature variation is relatively small and does not influence the cumulative radiation damage. Moreover, temperature-induced variations are reversible, whereas the measured resistance drift persists after irradiation. All post-irradiation electrical measurements were performed at room temperature under same laboratory conditions, ensuring consistent comparison before and after exposure.

For gamma ray irradiation, displacement damage in silicon is negligible, whereas ionization-induced loss in carrier concentration and carrier mobility change can influence electrical stability. Therefore, the experiments focused on evaluating post-irradiation changes in the resistance rather than instantaneous signal fluctuations during exposure.

Several studies examined MEMS accelerometers and pressure transducers using silicon piezoresistors intended for use in nuclear reactor environments. Those works reported an increase in resistance of the piezoresistive elements, which was attributed to the expansion of the depletion region surrounding the resistors, effectively reducing the current-carrying cross-sectional area. Similar trends have been observed in non-MEMS silicon piezoresistive elements [20]. However, the previous efforts were conducted under high-dose conditions, typically far above the operational levels encountered in practical robotic applications. In contrast, the present work focuses on the low-dose-rate regime, where the absorbed dose and dose rate are equivalent to those expected in decommissioning tasks outside the reactor.

## 3. Results and Discussion

### 3.1. Resistance Change After Irradiation

The resistance of ultra-thin piezoresistive Si sensors was tested at five different exposure times. The measured device resistance change was described in Equation (1). At zero mechanical load with no change in geometry, a small change in resistance reflects a change in resistivity:

(6) $${\left(\frac{∆\rho }{\rho }\right)}_{\varepsilon =0}=\frac{∆R}{R}$$

Meaning that the observed resistance drift directly represents irradiation-induced changes in resistivity, which is the static response of the silicon sensor to the radiation.

Overall, resistance changes are small at all doses tested, as summarized in Table 3. For specimens irradiated for 5.5 h, 17.77 h, and 28.72 h, a slightly observable increase in resistance of about 10~20 Ω was observed, relating to the 0.485~1.289% resistivity increase. Although the offset is small compared with the total resistance, it is non-negligible for producing a small apparent strain error in long-term use. In a doped silicon layer, the resistivity is determined by the carrier concentration n or p and carrier mobility μ:

(7) $$\rho =\frac{1}{qn\mu }$$

Taking a differential form

(8) $$\frac{∆\rho }{\rho }=-\frac{∆n}{n}-\frac{∆\mu }{\mu }$$

Irradiation-induced resistivity variation in doped silicon can arise from two microscopic effects: a change in the free-carrier density, and a change in carrier mobility caused by additional scattering from trapped charge or radiation-induced defects. γ-rays interact mainly via ionization rather than significant displacement damage. Prior studies show that even without significant displacement damage, ionization can alter charge states and local potentials, leading to additional carrier scattering and reduced mobility in silicon [13].

A quantitative comparison between the donor concentration and the radiation-induced defect density could be established from the absorbed dose. The phosphorous doping concentration in the active layer is on the order of $10^{20}$ cm^−3^. The corresponding number of generated electron-hole pairs per unit volume is

(9) $$N_{eh}=\frac{U}{E_{pair}}=\frac{D\cdot \rho_{Si}}{E_{pair}}$$

where U is the deposited energy density per volume, D is the absorbed dose, $\rho_{Si}$ is the density of silicon, $E_{pair}\approx 3.6\;eV$ is the average energy required to create one electron-hole pair in silicon.

For a total absorbed dose at level of 10 Gy, the estimated $N_{eh}$ is on the order of $10^{17}$ cm^−3^, which remains three orders of magnitude lower than the phosphorous doping concentration. Thus, the relative change in majority carrier concentration Δn/n is expected to be very low [25]. The observed resistance increase of lower than 1% is thus more naturally attributed to a slight degradation of the electron mobility, caused by ionization-induced traps and local potential fluctuations that enhance carrier scattering.

**Figure 5:**
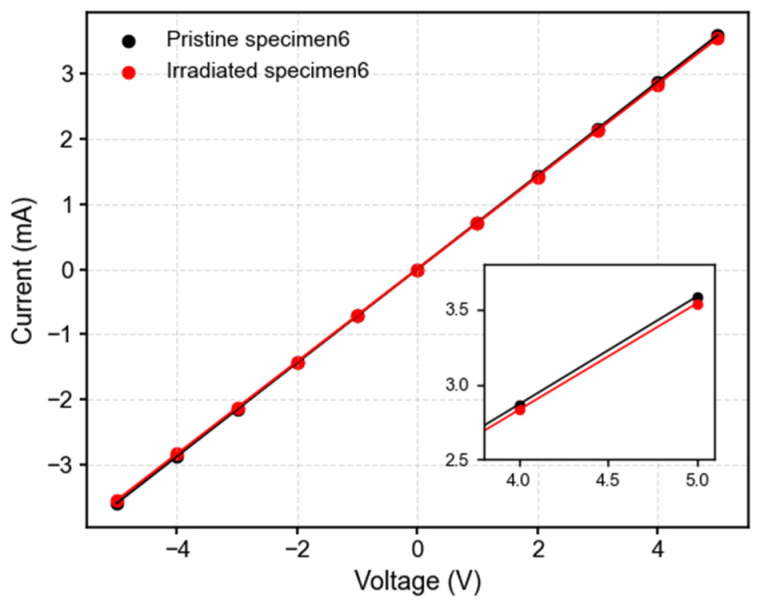
The comparison of IV characteristics of specimen 6 before and after irradiation.

To further verify these trends, current–voltage (IV) measurements were performed on the silicon specimen using a Keysight B1500A Semiconductor Device Analyzer. The applied bias was swept from −5 V to +5 V at room temperature to evaluate changes in electrical conductivity before and after irradiation. Figure 5 illustrates that IV characteristics remain strictly linear before and after irradiation, confirming that the contacts remain ohmic. And the silicon devices exhibited a slight reduction in slope of the IV curve after γ-ray exposure, consistent with a modest increase in resistivity. Taken together, these results suggest that low-dose γ-ray irradiation introduces only minor mobility-related perturbations in heavily doped silicon.

### 3.2. Gauge Factor Change After Irradiation

**Figure 6:**
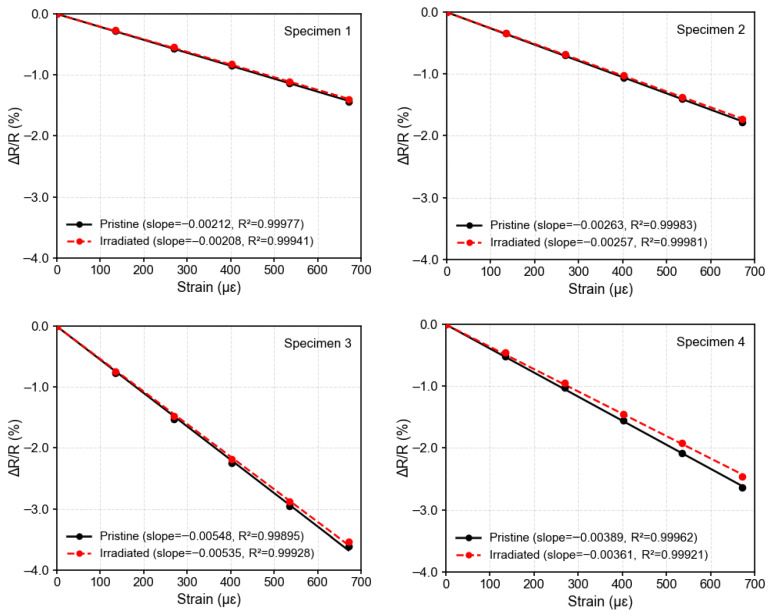

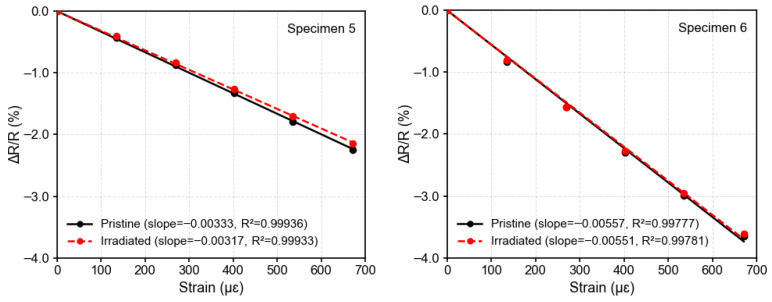
Fractional resistance change (ΔR/R) versus applied strain in n-type Si; lines indicate linear fits.

The resistance of each silicon piezoresistor was measured as a function of applied strain. Figure 6 shows the fractional resistance change (ΔR/R) plotted against applied strain for ultra-thin silicon gauges before and after γ-ray exposure. In both cases, the ΔR/R_0_–strain relationship remains highly linear (R^2^ > 0.99), confirming that irradiation at this dose does not disrupt the fundamental linearity of the piezoresistive response. Zhu et al. also demonstrate that result [19].

Despite identical fabrication parameters, variations in the initial sensitivity to the strain were observed among specimens. The measured gauge factors before irradiation ranged from approximately 20 to 60, as shown in Figure 2d. It reflects process related differences such as doping concentration [26], and strain-transfer efficiency through the adhesive interfaces. These factors influence the effective carrier density and mechanical coupling, resulting in modest baseline sensitivity dispersion. Because the purpose is to observe the difference caused by the radiation, the initial sensitivity of specimens is not necessarily required to be the same.

After γ-ray irradiation, several silicon strain gauges exhibited a slight reduction in the gauge factor. Specimens exposed for 17.77 h and 28.72 h exhibited noticeable reductions in gauge factor, with decreases of about 7.2% in specimen 4 and 4.8% in specimen 5. The polyimide substrate used in this study is Xenomax (Toyobo Co., Ltd., Osaka, Japan), a high-temperature-resistant film designed for mechanically stable flexible electronic applications. At the investigated dose level, the deposited energy is insufficient to cause chain scission or crosslinking that would modify the modulus or Poisson’s ratio of the polymer matrix [4]. Furthermore, the polyimide layer is only 5 μm thick and firmly bonded to a stainless-steel substrate. Therefore, the radiation-induced modification of the polyimide mechanical properties can be reasonably neglected.

To address the variability in initial device performance, a normalized comparison was performed by expressing the post-irradiation values relative to the initial state of each specimen. After normalization, it can be observed that, except for specimen 4 which shows a relatively larger deviation (~7.2%), the gauge factor variation of the remaining specimens is confined within approximately 5%. In addition, the resistance change across all specimens remains within 1.3%.

From the normalized plots, no clear systematic trend with increasing dose is observed within the investigated dose range. However, the overall variation trends of resistance and gauge factor remain consistent with each other, both exhibiting small and comparable changes within a narrow range.

It should be noted that fabrication-related variations, such as differences in doping concentration or implantation profiles, may contribute to slight differences in the magnitude of radiation-induced changes among specimens. However, such process-induced variations are not expected to significantly alter the overall trend of the response, as the underlying degradation mechanism remains governed by similar carrier transport and scattering effects under gamma irradiation.

### 3.3. Mechanism Analysis of Gauge Factor Degradation

To further understand the experimentally observed degradation in gauge factor, a theoretical analysis based on carrier transport mechanisms is presented. To interpret the gauge factor change, the measured gauge factor K can be expressed as

(10) $$K=1+2\nu +\frac{1}{\varepsilon }\frac{d\rho }{\rho }$$

where ν is Poisson’s ratio, n the carrier concentration, and μ the carrier mobility. Combined with Equation (8), the gauge factor K could be obtained as

(11) $$K=\left(1+2\upsilon \right)-\frac{1}{\varepsilon }\left(\frac{\Delta n}{n}+\frac{\Delta \mu }{\mu }\right)$$

indicating that changes in K arise from variations in carrier concentration (n) and mobility (μ) under strain.

However, the observed degradation in the gauge factor under gamma ray irradiation cannot be explained by a simple decrease in mobility. In n-type silicon, the piezoresistive effect is largely governed by the valley transfer model, proposed by C. S. Smith [27]. Electrons occupy six equivalent valleys in the conduction band. When under tensile strain, strain lowers the energy of the longitudinal valleys and modifies the conductivity effective mass, leading to a strain-induced mobility enhancement. The mechanism is different from the piezoresistance in p-type silicon, which was analyzed based on hole transfer and conduction mass shift caused by stress [28].

Under the gamma ray ionization, the carrier concentration is expected to remain essentially unchanged. The experimentally observed increase in resistance and reduction in gauge factor are therefore more naturally attributed to changes in the mobility term. The ionization-caused extra scattering slightly reduces overall mobility and dilutes the fraction of scattering that is strain-dependent. From a transport perspective, the total mobility can be interpreted by Matthiessen’s rule [29], in which the inverse mobility is written as the sum of contributions from different scattering mechanisms:

(12) $$\frac{1}{\mu_{total}}=\frac{1}{\mu_{lattice}}+\frac{1}{\mu_{impurity}}+\frac{1}{\mu_{defect}}$$

Here, $\mu_{lattice}$ is limited by phonon scattering, $\mu_{impurity}$ by ionized dopants, and $\mu_{defect}$ by radiation ionization-induced defects. The total mobility is the sum of a strain-sensitive component and strain-insensitive component. The presence of this additional component reduces the magnitude of (1/ε) Δμ/μ, which appears experimentally as a modest decrease in the gauge factor. Nagarajan et al. demonstrated that modulation doping suppresses ionized impurity scattering centers, leading to an increase in carrier mobility [25]. In contrast, gamma irradiation introduced defects-related scattering term that is insensitive to strain. The additional scattering reduces the relative contribution of strain-dependent mobility modulation.

### 3.4. Strain Error Evaluation

Although γ irradiation introduces a baseline resistance offset, such offsets can be effectively eliminated by normalizing the signal using the fractional resistance change ΔR/R_0_. This normalization strategy allows strain estimation to be insensitive to absolute resistance drift. After normalization, the dominant radiation-induced error source is the change in gauge factor, which alters the strain-to-signal transduction pathway.

**Figure 7:**
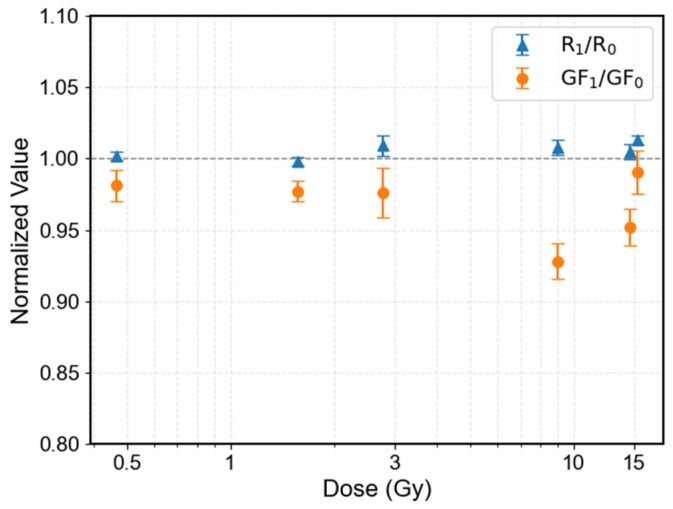
Normalized resistance change and gauge factor change as a function of accumulated gamma ray dose. All values are normalized to the initial state of each specimen.

**Figure 8:**
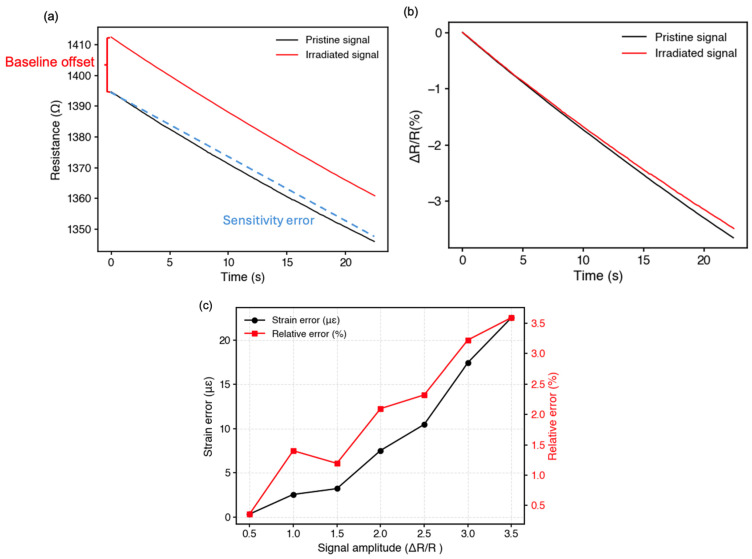
(**a**) The raw data of sensor under increasing strain loading; (**b**) the normalized signal to ignore the original offset signal; (**c**) the strain error and relative error upon each signal amplitude.

Based on the normalized signal analysis, the radiation-induced gauge factor degradation results in a maximum strain estimation error of approximately 22.5 µε within the tested operating range (<700 µε), corresponding to a relative error of about 3.5%. Figure 7 illustrates the procedure used to quantify the impact of radiation-induced electrical drift on strain estimation accuracy. The real-time resistance signal in Figure 8a exhibits a clear offset shift and a slightly reduced slope after irradiation. The normalization in Figure 8b removes the influence of baseline offset and isolates the strain-dependent component of the signal. Figure 8c quantifies the strain estimation error arising from this sensitivity change. Taking the normalized signal amplitude (ΔR/R) as the feedback variable used by the robotic control system, the strain inferred from the irradiated sensor output is compared with the true applied strain calibrated before irradiation. The resulting difference is defined as the strain error. Importantly, the high degree of linearity (R^2^ > 0.99) observed after irradiation ensures a predictable strain–signal response. This allows for efficient recalibration and gain compensation, as the sensor’s behavior remains free from significant nonlinear distortion.

It should be noted that the above analysis is based on γ-ray irradiation, while ionization effects are dominant. Under such conditions, previous studies have shown that ionization-induced degradation has a relatively minor effect on the mechanical response of silicon-based sensors. However, in environments containing high-energy particles, displacement damage in the silicon lattice may become significant. The introduced displacement damage could significantly affect both electrical and mechanical responses. In such cases, additional radiation-hardened design or radiation-resistant packaging strategies may be required [30].

For robotic manipulation tasks, tactile feedback plays a vital role in regulating prehensile forces and ensuring stable grasps [31]. While ideal tactile sensors aim for high linearity and low hysteresis, practical applications frequently operate in the presence of sensor noise and non-ideal responses. Reviews on both dexterous hands and humanoid skins indicate that strict requirements on absolute analytical precision can often be relaxed when the primary goal is detecting slip or monitoring discrete sensory events to track state transitions [32]. These transitions provide the necessary updates for internal models to optimally adjust grip forces. According to Table 4, the maximum strain estimation error of approximately 3.5% observed in this study does not compromise grasp stability or manipulation performance.

## 4. Conclusions

Ultra-thin silicon piezoresistive sensors were irradiated with a gamma ray dose rate of 0.5 Gy·h^−1^ (air) to evaluate their electrical output. The number of γ–matter interaction events in the silicon volume is extremely sparse. This is consistent with the observed resistance change, which increased by at most 1.289% after irradiation. No thermal annealing was applied after exposure, and those small offsets persist in repeated measurements, indicating quasi-permanent resistivity change.

The gamma ray radiation ionization effect on the dynamic response of the strain gauges is detectable. Ultra-thin silicon strain gauges irradiated for 9.001 Gy and 14.565 Gy exhibit a reduction in gauge factor. And for strains greater than 600 μm, a deviation in the differential resistance change was observed. This behavior may result from strain-insensitive scattering mechanisms that reduce the strain-dependent mobility modulation in the ultra-thin conductive layer.

Despite these small drifts, the strain–resistance response of all silicon gauges remains highly linear (R^2^ > 0.99) before and after irradiation. This indicates that the fundamental coupling between strain and resistivity has not been affected by the low-dose γ exposure. At higher strain levels, the deviations from the pristine response may become more pronounced, but within the strain range tested here, the linear mapping from strain to electrical signal is preserved.

From an application standpoint, the results suggest that ultra-thin n-type silicon strain gauges retain mechanical robustness and electrical linearity under gamma irradiation at dose rates relevant to nuclear decommissioning. The radiation-induced changes manifest as slow offsets in baseline resistance and a modest reduction in piezoresistive sensitivity rather than abrupt failure. Provided that such offsets and sensitivity drifts are accounted for through calibration and appropriate signal processing, the studied sensors are suitable candidates for long-duration tactile and force sensing in γ-dominated environments.

## Figures and Tables

**Table 2:** The thickness and material modulus of each layer.

Layer	Material	Thickness $d_{i}$ [mm]	Top Coordinate $h_{i}$ [mm]	Young’s Modulus $E_{i}$ [Pa]
1	SUS304 (Cantilever)	0.500	0.500	$1.93\times 10^{11}$
2	CC-33A (Adhesive)	0.01	0.5	$1.00\times 10^{9}$
3	Polyimide (Support film)	0.005	0.510	$8.80\times 10^{9}$
4	Silicon (Strain gauge)	0.005	0.515	$1.80\times 10^{11}$

**Table 3:** Resistance of five different ultra-thin Si piezoresistive sensors before and after Co-60 γ-irradiation at different exposure times.

Sample	Exposure Time (h)	Irradiation Dose (Gy)	Pristine Resistance (Ω)	Resistance After Irradiation (Ω)	Resistance Change (%)
Specimen 1	0.92	0.465	1272.48	1274.59	+0.165
Specimen 2	3.1	1.572	1268.59	1269.12	+0.042
Specimen 3	5.5	2.781	1443.78	1456.24	+0.863
Specimen 4	17.77	9.001	2667.18	2687.05	+0.745
Specimen 5	28.72	14.565	2051.70	2061.65	+0.485
Specimen 6	28.72	14.565	1394.55	1412.53	+1.289

**Table 4:** Strain error and error percentage at different signal amplitudes.

Amplitude	Strain Error (με)	Relative Error (%)
0.5	0.34	0.36
1	2.56	1.40
1.5	3.21	1.19
2	7.53	2.10
2.5	10.47	2.32
3	17.44	3.22
3.5	22.54	3.59

## Data Availability

Data of this research is available upon request via first author.
